# Repair at Single Targeted DNA Double-Strand Breaks in Pluripotent and Differentiated Human Cells

**DOI:** 10.1371/journal.pone.0020514

**Published:** 2011-05-25

**Authors:** Hua Fung, David M. Weinstock

**Affiliations:** Department of Medical Oncology, Dana-Farber Cancer Institute, Harvard Medical School, Boston, Massachusetts, United States of America; University of Massachusetts Medical School, United States of America

## Abstract

Differences in *ex vivo* cell culture conditions can drastically affect stem cell physiology. We sought to establish an assay for measuring the effects of chemical, environmental, and genetic manipulations on the precision of repair at a single DNA double-strand break (DSB) in pluripotent and somatic human cells. DSBs in mammalian cells are primarily repaired by either homologous recombination (HR) or nonhomologous end-joining (NHEJ). For the most part, previous studies of DSB repair in human cells have utilized nonspecific clastogens like ionizing radiation, which are highly nonphysiologic, or assayed repair at randomly integrated reporters. Measuring repair after random integration is potentially confounded by locus-specific effects on the efficiency and precision of repair. We show that the frequency of HR at a single DSB differs up to 20-fold between otherwise isogenic human embryonic stem cells (hESCs) based on the site of the DSB within the genome. To overcome locus-specific effects on DSB repair, we used zinc finger nucleases to efficiently target a DSB repair reporter to a safe-harbor locus in hESCs and a panel of somatic human cell lines. We demonstrate that repair at a targeted DSB is highly precise in hESCs, compared to either the somatic human cells or murine embryonic stem cells. Differentiation of hESCs harboring the targeted reporter into astrocytes reduces both the efficiency and precision of repair. Thus, the phenotype of repair at a single DSB can differ based on either the site of damage within the genome or the stage of cellular differentiation. Our approach to single DSB analysis has broad utility for defining the effects of genetic and environmental modifications on repair precision in pluripotent cells and their differentiated progeny.

## Introduction

The preservation of genomic integrity requires the recognition and repair of a vast array of DNA damage, including strand breaks and chemical base modifications. DNA double-strand breaks (DSBs) are particularly challenging to repair, as neither strand remains intact to template repair for the other. DSB repair in mammalian cells either utilizes a homologous template or involves nonhomologous end-joining (NHEJ). The “classical” pathway of NHEJ, which is essential for lymphocyte antigen receptor rearrangements and ionizing radiation resistance, is mediated by the DNA end-binding heterodimer KU70/KU80, the kinase DNA-PKcs, the XRCC4/XLF/LIG4 ligase complex, and the endonuclease Artemis [1], [2].

DSB repair that utilizes a homologous template can either involve homologous recombination (HR) or single-strand annealing (SSA) [3]. In both pathways, the DSB end is processed to a single-strand 3′ tail. In HR, the single-strand tail undergoes RAD51-dependent invasion of a homologous duplex followed by template-dependent synthesis. HR is generally considered to be a precise form of repair, because it can restore the original sequence if the sister chromatid or another identical sequence is used as a template [4]. HR can be mutagenic if the template is similar but not identical to the broken sequence. For example, HR between homologous chromosomes can result in loss of heterozygosity. SSA, in contrast with HR, involves the annealing of sequence repeats located near the DSB. SSA is always mutagenic, as the sequence between the repeats is deleted. SSA has different genetic requirements from HR and does not involve strand invasion [5].

The balance between DSB repair pathways is a key determinant of repair precision, and appears to differ between cell types and during different phases of the cell cycle [6]. HR is most active during the late S and G2 phases, when the sister chromatid is available to template repair. NHEJ predominates in G0 and G1, when HR could promote loss of heterozygosity, but remains active throughout the cell cycle [2]. At least to some extent, the pathways are competitive. For example, loss of classical NHEJ factors promotes HR at an endonuclease-mediated DSB [7]. Similarly, loss of NHEJ proteins can restore homologous recombination and mitomycin C resistance in cells lacking HR factors [8], [9], [10].

Stem cells, including embryonic stem cells, have been utilized in studies of DNA repair as they can be propagated in culture and lack the genetic alterations present in cancer cells [11]. Previous studies that characterized DSB repair within both human embryonic stem cells (hESCs) and somatic stem cells have primarily utilized nonspecific clastogens, such as ionizing radiation (IR), to examine effects on survival and cell cycle arrest, as well as the efficiency of repair and the induction of gross chromosomal rearrangements [12], [13], [14], [15]. This approach has several shortcomings. First, even low doses of nonspecific clastogens will induce scores of DSBs within each cell. Under normal circumstances, a stem cell *in situ* would not experience such extensive simultaneous damage, so arguments about the implications of damage response in this context are tenuous. Second, hESCs irradiated with doses as low as 2 Gy undergo ATM-dependent cell cycle arrest in the G2 phase [14], [16], which may favor the repair of DSBs by HR. Third, exogenous clastogens like radiation or alkylating agents can produce chemical base alterations that require end modification prior to ligation. In contrast, DSBs that result from endogenous processes, such as cleavage by topoisomerase II or replication fork collapse, lack DNA adducts and other alterations, and may be either directly ligated or serve as intermediates for HR. Finally, repair efficiency (*i.e.*, the rapidity of repair at multiple DSBs) and repair precision (*i.e.*, repair without sequence alteration) are not necessarily correlated.

Of great importance, differences in *ex vivo* cell culture conditions can drastically affect stem cell physiology. These differences include propagation in the presence or absence of feeder cells [17], under conditions of hypoxia [18], or with uniquely conditioned media [19], [20], [21]. As so many variables could potentially alter genetic instability *ex vivo*, we sought to establish an assay for measuring the effects of chemical, environmental, and genetic manipulations on the precision of repair at a single DSB. Such an assay would have widespread utility for optimizing culture conditions that maintain genetic integrity during cell propagation, modification and/or differentiation. To this end, we developed an approach for site-specific targeting of a single DSB in human cells within the context of a DNA repair reporter. This allows for the absolute quantification of imprecise repair by HR, NHEJ and SSA, which can be compared across various culture conditions, stages of differentiation, genetic backgrounds or genomic locations. For the first time, we demonstrate that the phenotype of repair at a single DSB differs across isogenic hESCs based on either the site of the DSB within the genome or the stage of cellular differentiation.

## Results

### DSB repair in hESCs

**Figure 1:**
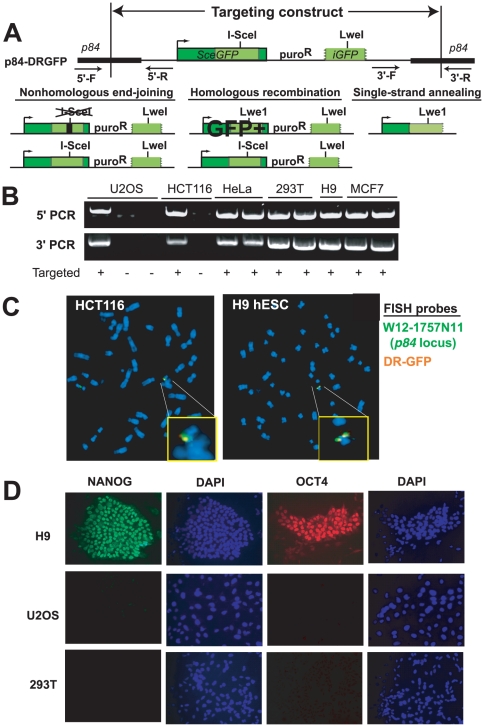
Targeting of p84-DR-GFP. **A**. A DSB formed by I-SceI within the *SceGFP* gene in p84-DR-GFP can either be repaired by nonhomologous end-joining, homologous recombination or single-strand annealing. HR using the *iGFP* template establishes a functional *GFP*. Precise NHEJ or HR using the *SceGFP* on the sister chromatid as a template can reestablish the I-SceI site. Arrows indicate primers for confirming targeting to *p84*. **B**. PCR demonstrates targeting at the 5′ and 3′ borders of p84*-*DRGFP. **C**. FISH demonstrates overlap of the *p84* locus (green) and DR-GFP (orange) probes indicating targeting of DRGFP to the *p84* locus in hESCs and HCT116 cells. Insets are magnified images of the indicated regions. **D**. Immunofluorescence microscopy for pluripotency markers in H9 hESCs, U20S and 293T cells.

To assay the phenotype of repair at a single DSB, we utilized the Direct Repeat (DR)-GFP reporter developed within the Jasin laboratory [7]. DR-GFP has been widely utilized in a broad range of mammalian cell lines to estimate the relative frequencies of NHEJ, HR and SSA after cleavage at a single recognition site [22]. DR-GFP (Figure 1A) includes a full-length GFP gene (*SceGFP*) disrupted by a recognition site for the I-SceI endonuclease, which cleaves a nonpalindromic 18 bp sequence that is not present in the human or mouse genomes. After I-SceI cleavage, HR using a downstream repeat (*iGFP*) to template repair replaces the I-SceI recognition site with an LweI site and produces a functional *GFP* that can be detected by flow cytometry. Repair by SSA also replaces the I-SceI recognition site with a LweI site but does not generate a full-length GFP (Figure 1A). Thus, HR using the *iGFP* template, SSA or imprecise NHEJ results in “loss” of the I-SceI recognition site.

HR that results in a functional *GFP* could utilize the *iGFP* template on either the same chromatid that is cleaved by I-SceI or the sister chromatid. HR could also utilize the *SceGFP* on the sister chromatid, if it is not concurrently cleaved by I-SceI. This would result in reestablishment of the I-SceI site and no genetic evidence that the HR took place. Thus, GFP-positive frequency is a surrogate for the frequency of HR, rather than an absolute measure of the total number of HR events. Similarly, precise repair by NHEJ of the I-SceI break without sequence modification can restore the I-SceI recognition site. A restored site leaves no genetic evidence of the cleavage and repair event, but becomes a substrate for subsequent cycle(s) of cleavage and repair [23].

To avoid position effects, we made use of targeted gene addition with zinc finger nucleases (ZFNs). ZFNs are fusions of the Fok1 nuclease domain and a DNA recognition domain composed of engineered C2H2 zinc-finger motifs [24]. Upon binding of two fusion proteins in inverse orientation, dimerization of the nuclease domains results in site-specific cleavage. Repair of the resulting DSB by synthesis-dependent strand annealing using an introduced template can result in gene targeting at high efficiency [25], [26]. We utilized a recently described ZFN set that allows for efficient and specific targeted integration into the *p84* (AAVS1/PPP1R12C) locus to generate a panel of isogenic lines with DR-GFP targeted to that position [27]. *p84* is considered a “safe-harbor” locus for integrating transgenes, as it is constitutively expressed across a variety of cell types and biallelic disruption results in no discernible phenotype [28], [29].

**Figure 2:**
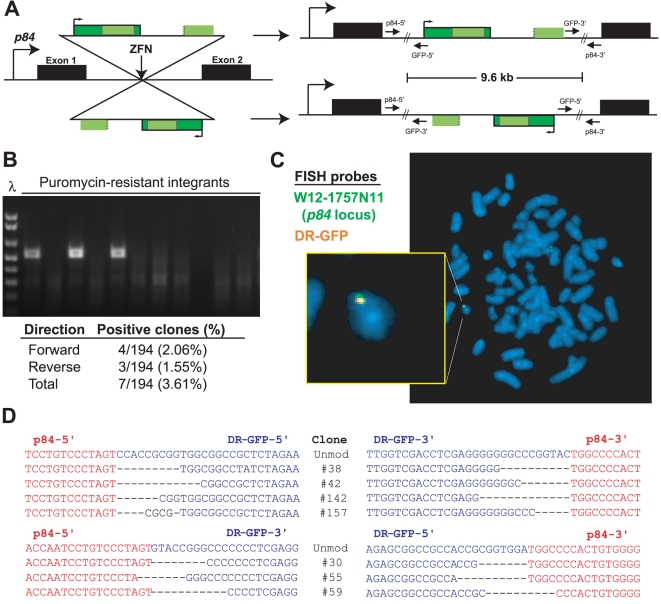
Site-specific targeting without sequence homology. **A**. DR-GFP can be integrated at the *p84* locus in either orientation. **B**. PCR with p84-5′ and GFP-3′ primers demonstrates three 293T clones with site-specific integration. λ indicated DNA ladder. **C**. FISH demonstrates colocalization of probes against DR-GFP (green) and spanning the *p84* locus (orange) in 293T cells. **D**. Sequences at 5′ and 3′ borders of integrated DR-GFP. Red sequence is from the *p84* locus and blue sequence is from the DR-GFP construct. Integration with no sequence modification is indicated as ‘unmod’.

To target DR-GFP, we first attempted an NHEJ-based gene capture strategy. The HPRT-DRGFP reporter [7], which contains DR-GFP flanked by sequence from the murine *Hprt* gene was linearized and co-transfected with ZFNs targeting the *p84* locus [27] in 293T cells. No sequence homology is present between HPRT-DRGFP and *p84*, so integration results solely through NHEJ. Integrants were analyzed by PCR and fluorescence *in situ* hybridization (FISH) for targeting (Figure 2). Overall, 7 (3.6%) of 194 integrants had undergone site-specific targeting, including 4 in the same orientation as *p84* coding sequence and 3 in the opposite orientation (Figure 2). Thus, even in the absence of any sequence homology, targeting of the 9.6 kb DR-GFP sequence can be performed through ZFN-directed NHEJ.

To increase the targeting efficiency, we generated the p84-DRGFP reporter, which contains the DR-GFP transgene flanked by 0.8 kb arms identical to intron 1 of *p84* (Figure 1A) [27]. Previous studies demonstrated efficient targeting of a smaller cassette to *p84* in hESCs utilizing the same flanking arms and ZFNs [27], [30], [31]. We co-transfected p84-DRGFP with the p84 ZFN expression vector into a panel of human cell lines, including the hESC lines H9 and BG01. Integrants were isolated from single cell-derived colonies by selection in puromycin. hESCs were maintained on Matrigel-coated plates in the absence of feeder cells. The presence of a single integrant was determined by quantitative PCR (data not shown). Targeting was identified by PCR (Figure 1B) in 60–80% of puromycin-resistant clones across all cell lines. Single copy integration and *p84* locus targeting were confirmed by FISH (Figure 1C). Expression of pluripotency markers was confirmed in hESC clones containing p84-DRGFP by immunofluorescence microscopy (Figure 1D).

### hESCs have very low rates of HR using the *iGFP* template

**Figure 3:**
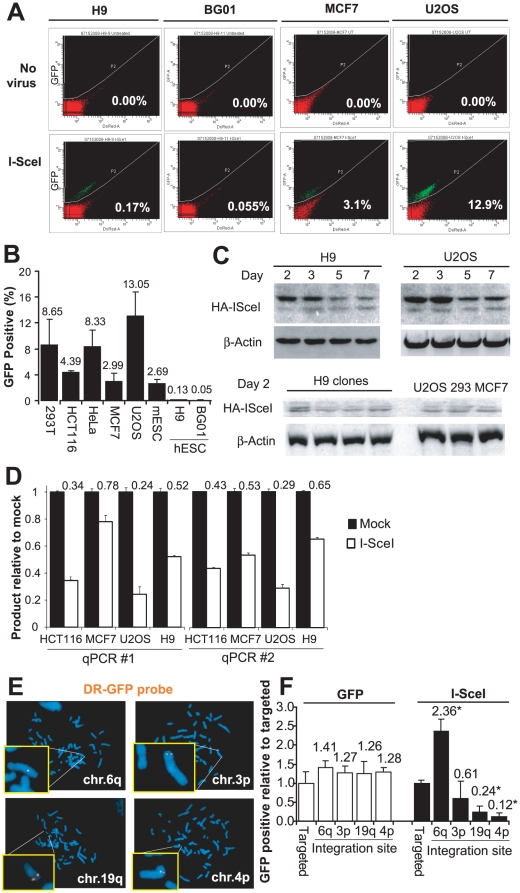
HR of I-SceI-induced DSBs in p84-DR-GFP. **A**. Flow cytometry before or 96 hours after transduction with an I-SceI-expressing lentivirus. Values represent percent of GFP-positive cells. **B**. Frequency of GFP-positive cells by flow cytometry 96 hours after I-SceI transduction in human cell lines, mouse embryonic stem cells (mESC) and two hESC lines. Error bars indicate one standard deviation. **C**. Immunoblotting with anti-HA antibodies at various time points after I-SceI transduction. **D**. Two different sets of qPCR primers were used to amplify the region flanking the I-SceI site using genomic DNA isolated from cells 24 hours after mock transduction or transduction with I-SceI. The reduction in the amount of amplified product compared to mock transduction represents the fraction cleaved by I-SceI. **E**. Localization of nontargeted DR-GFP in hESC clones by FISH using a probe derived from DR-GFP (orange). **F**. Frequency of GFP-positive cells by flow cytometry among hESC clones with non-targeted DR-GFP relative to hESC cells with *p84* targeted DR-GFP after transduction of GFP or I-SceI. * indicates p<0.01 compared with *p84* targeted DR-GFP.

To estimate the frequency of I-SceI-induced HR, we transduced I-SceI into cells containing targeted p84-DRGFP. After 96 hours, we measured the frequency of GFP+ cells by flow cytometry. Strikingly, the frequency of GFP+ cells was 20–260-fold lower among hESCs compared to somatic cell lines and murine ESCs (mESCs) that each contain a single integrated copy of DR-GFP (Figure 3A, 3B). The extent of fold reduction observed between hESCs and the other cell types is similar to that seen from the loss of HR factors like BRCA2 [32]. The frequency of GFP-positive mESCs after I-SceI expression (Figure 3B) was similar to previous reports with these cells [7].

Differences in the expression of I-SceI across cell types could explain the low frequency of GFP+ hESC recombinants. However, immunoblotting after I-SceI transduction demonstrated similar expression in hESCs and somatic cell lines at multiple time points after transduction (Figure 3C).

Even with similar I-SceI protein expression, it is possible that cutting is somehow less efficient in hESCs. To address this, we used two different sets of qPCR primers (Table 1) to amplify the sequence overlapping the I-SceI site. With this approach, the fraction of cells with cleaved I-SceI at a given time point is equivalent to the percent reduction in qPCR product compared with untransduced control. Using both primer sets, the percent of cells with cleavage at the I-SceI site 24 hours after transduction in hESCs fell in the same range as the somatic human cell lines (Figure 3D). We assayed cleavage 24 hours after transduction to ensure that I-SceI was maximally expressed and to minimize the number of cells that had undergone imprecise repair and thus could not be cleaved. The percent of cells cleaved at any given time point is an imperfect measure of cleavage efficiency, as the efficiency of repair will also affect the likelihood that any given cell will be cleaved at a single time point. Nonetheless, similar findings using the qPCR strategies across cell lines suggests that the difference in frequency of GFP-positive cells between hESCs and somatic cell lines does not result from a drastic difference in I-SceI cutting.

**Table 1:** PCR and qPCR primers.

Amplicon		Primers (Forward then Reverse)	
I-SceI or LweI site-loss		5′- AGGGCGGGGTTCGGCTTCTGG 5′- CCTTCGGGCATGGCGGACTTGA	
Reporter copy number for quantitative PCR		5′- CGGCGCCGGCAGGAAGGAA 5′- CTCTAGAGCCGCCGGTCACACG	
Reference amplicon from *APE1* on chr.14 for quantitative PCR		5′- CGGCACGCGTGGGATGAA 5′- GCCTTGGCGCTCTTGTGG	
Nested PCR to determine I-SceI cleavage efficiency	1^st^ round, PCR ×18 cycles	5′- AGGGCGGGGTTCGGCTTCTGG 5′- CCTTCGGGCATGGCGGACTTGA	
	2^nd^ round, quantitative PCR	5′- GACGTAAACGGCCACAAGTT 5′- AAGTCGTGCTGCTTCATGTGor5′- GCAACGTGCTGGTTATTGTG 5′- TGCCTCGTGGGTCTTCTACT	
Reference amplicon from near *p84* on chr.19 for PCR of I-SceI cleavage efficiency	1^st^ round, PCR ×18 cycles	5′- TTCTGTGCTGGGGTAGAACC 5′- CCAAACCCCAGTCCTCTACA	
	2^nd^ round, quantitative PCR	5′- CTCAAACTGCATGGCTCAAA 5′- CCAAACCCCAGTCCTCTACA	
Confirming HPRT-DRGFP targeting at *p84* without homologous arms	Same orientation as *p84* coding	5′ junction	5′-AGGATCCTCTCTGGCTCCAT 5′-CGAGATCTGATGCCCTCTTC
		3′ junction	5′-TGTAGAGGACTGGGGTTTGG 5′-CTTGCTTTCTTTGCCTGGAC
	Opposite orientation	5′ junction	5′-CAGCTCAGGTTCTGGGAGAG 5′-TGTAGAGGACTGGGGTTTGG
		3′ junction	5′-GCAAACATGCTGTCCTGAAG 5′-TTGCTCTCTGCTGTGTTGCT

### Site of integration affects the frequency of HR

The advantage of utilizing a targeted reporter across multiple cell lines is the avoidance of potential locus-specific effects on DSB repair. To determine whether genomic location of the single DSB truly affects repair phenotype, we analyzed four additional H9 hESC clones that integrated a single copy of DR-GFP at other sites in the genome, as demonstrated by FISH (Figure 3E).

After expression of I-SceI, the frequency of GFP+ cells varied significantly across lines, with a nearly 20-fold difference between the highest and lowest frequency clones (p<0.01; Figure 3F). In contrast, the frequency of GFP+ cells did not significantly differ after transduction with a GFP-expressing virus (Figure 3F), suggesting that the effect on frequency of HR is not due to differences in transduction efficiency across cell lines. Thus, the frequency of HR differs significantly across isogenic cells based solely on the site of cleavage within the genome.

### Imprecise NHEJ is reduced in hESCs

**Figure 4:**
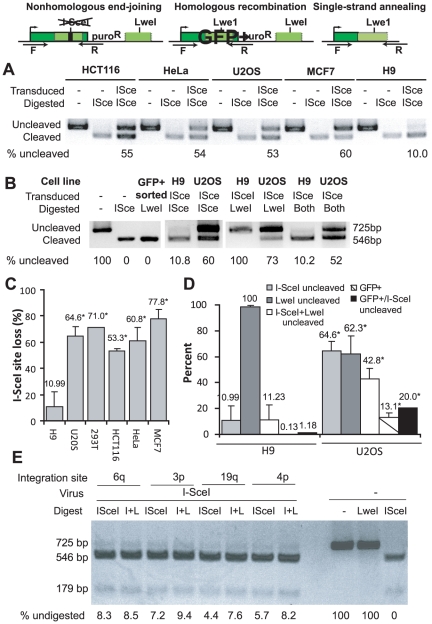
Repair through NHEJ, HR or SSA. **A**. Repair through imprecise NHEJ results in “loss” of the I-SceI site while repair through HR or SSA replaces the I-SceI site with an LweI site. DNA was isolated 96 hours after I-SceI transduction. After PCR amplification with the F and R primers and digestion with I-SceI, the uncleaved band (725 bp) represents product from cells that have undergone imprecise repair through any of the three pathways. **B**. Digestion of the PCR product with LweI generates a cleaved band (546 bp) that represents product from cells that have undergone HR or SSA. After digestion with both I-SceI and LweI (indicated as “Both”), the uncleaved band represents product from cells that have repaired by imprecise NHEJ. “GFP+” indicates an I-SceI transduced population that was sorted to be 100% GFP-positive, which served as a control for LweI digestion. **C**. Comparison of the fractions of uncleaved product after PCR amplification and I-SceI digestion. * indicates p<0.01 compared with H9. Error bars indicate one standard deviation. **D**. Comparison of the fractions of uncleaved product after digestion with the indicated enzymes. “GFP+” values are from flow cytometry after I-SceI transduction (Figure 3B). **E**. PCR product obtained from H9 hESC clones with non-targeted DR-GFP 96 hours after I-SceI transduction was digested with I-SceI, LweI or both (indicated as ‘I+L’). Untransduced cells are included to demonstrate complete digestion with I-SceI enzyme.

To determine the overall frequency of I-SceI site loss, we collected cells 4 days after I-SceI transduction and PCR amplified the region flanking the I-SceI site (Figure 4) [7]. The fraction of cells that had undergone sequence modification, either by HR, NHEJ or SSA, was measured by quantifying the fraction of PCR product that failed to digest with I-SceI (Figure 4A). The overall fraction of PCR product resistant to I-SceI cleavage (*i.e.,* the cumulative I-SceI site loss through NHEJ, HR and SSA) averaged 10.99% in H9 hESCs compared with 53–78% in somatic cell lines (Figure 4C; p<0.01 for hESCs versus HCT116, HeLa, U2OS or MCF7).

The overall rate of I-SceI site-loss was 5–7-fold less in hESCs compared with somatic cell lines, while the frequency of HR using the GFP template (as measured by percent GFP+) was 20–260-fold less. In other words, the fraction of I-SceI site loss that resulted from HR was only 1.18% for hESCs (0.13% GFP-positive/10.99% site loss) compared to 20.28% (13.1%/64.6%) for U2OS cells (Figure 4D). Thus, HR contributes a very small fraction of the overall I-SceI site loss in hESCs.

Next, we digested the PCR products obtained from H9 hESCs and U2OS cells with LweI, which will cleave amplicons that have repaired by either HR or SSA (Figure 4B). Approximately 38% of the PCR product from U2OS cells transduced with I-SceI was cleaved by LweI. In contrast, no LweI-cleaved product was detectable from hESCs after I-SceI transduction (the lower limit of detection is approximately 2%), indicating that site loss through both HR and SSA is infrequent in hESCs under these conditions.

To more precisely quantify the subset of I-SceI site-loss that resulted from NHEJ, we digested the PCR products with both I-SceI and LweI (Figure 4B), which cleaves all products except those repaired by imprecise NHEJ. Approximately 11% of the product from hESCs after I-SceI transduction was resistant to digestion with either I-SceI alone or I-SceI+LweI, confirming that essentially all I-SceI site loss in these cells is through NHEJ (Figure 4B). Very similar results were obtained from the four hESC clones with non-targeted integration of the DR-GFP reporter at different genomic sites (Figure 4E), indicating that the frequency of imprecise NHEJ is similar across different genomic locations. In contrast with the hESCs, LweI cleaved approximately one-third of the I-SceI undigested product from U2OS cells (Figure 4D; 42.87% I-SceI+LweI undigested versus 64.61% I-SceI undigested).

We sequenced individual imprecise NHEJ events from U2OS cells (n = 40) and hESCs (n = 36) (Table S1). There were no significant differences in sequence modifications observed at the repair junctions between the U2OS and hESC clones, including the frequency of insertions (30% vs. 17%), mean insertion length (3.7 bp vs. 2 bp), length of deletions (5.9 bp vs. 5.1 bp), and use of short stretches of overlapping microhomology (67.8% vs. 70% among clones lacking insertions) (Table S1). Thus, although imprecise NHEJ at a single DSB was significantly less common in hESCs, the phenotype of imprecise NHEJ was similar between the two cell types.

### DNA-PKcs contributes to repair of clastogen-induced damage in hESCs

**Figure 5:**
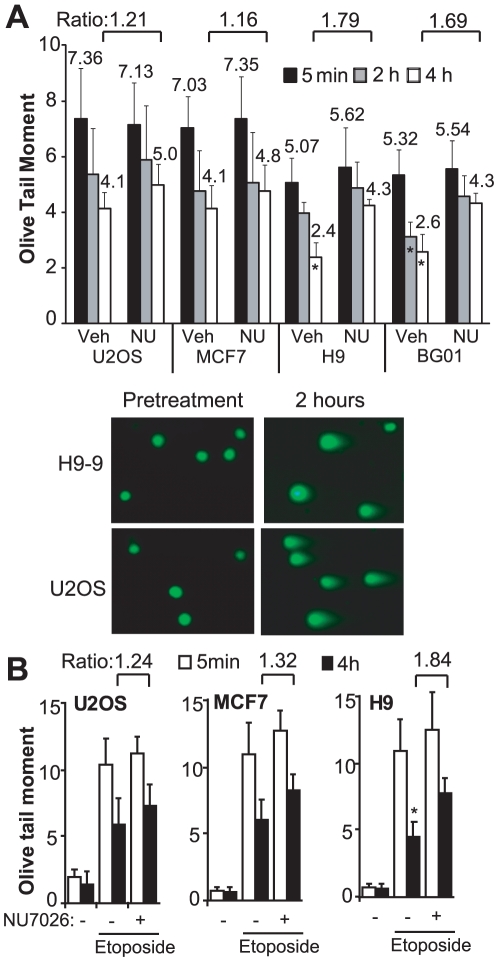
DNA-PKcs-dependent and -independent repair after low-dose IR. **A**. Neutral comet assay was performed after irradiation with 2 Gy in the presence of NU7026 (Nu) or vehicle (Veh) and the olive tail moment was calculated for at least 50 cells for each condition at the indicated time points. Error bars indicate one standard deviation. Indicated ratios are between olive tail moments for the same cell line in the presence of NU7026 or vehicle. * indicates p<0.01 compared with same cell line and time point treated with vehicle. **B**. Comet assays were performed after treatment with etoposide or vehicle (-).

Although site loss in hESCs was predominately mediated by NHEJ, this does not clarify the relative contributions of NHEJ and HR to overall repair, as precise repair of the I-SceI break by either NHEJ or by HR using *SceGFP* on the sister chromatid will reestablish the I-SceI site. To address this, we treated hESC, MCF7 and U2OS lines with 2 Gy of ionizing radiation, a dose which does not result in significant cell death but can promote G2 cell cycle arrest [14], [16]. At fixed time points, we measured the extent of persistent DNA damage using the neutral comet assay, which quantifies only DSBs (Figure 5A). The extent of damage induced by IR, as measured 5 minutes after irradiation, was reduced in hESCs compared with U2OS and MCF7 cells (Figure 5A). However, the kinetics of repair of IR-induced breaks were similar in hESCs, MCF7 and U2OS cells (Figure 5A). For example, by 4 hour after irradiation, the olive tail moment had decreased 44% in U2OS cells compared with 48–53% in the hESC lines (Figure 5A).

Banuelos et al. [33] reported that hESCs, in contrast with mESCs, express high levels of DNA-PKcs. To determine the contribution of DNA-PKcs to repair after low-dose IR, we treated cells with the selective DNA-PKcs enzymatic inhibitor NU7026 [34] before and after irradiation. As expected, NU7026 had no effect on the overall extent of damage induced by IR, as measured by the olive tail moment 5 minutes after irradiation (Figure 5A). NU7026 had a slight effect on repair in U2OS and MCF7 cells, with an increase in olive tail moment of 1.21-fold and 1.16-fold, respectively, at 4 hours after irradiation (Figure 5A). The effect was greater in hESCs, with 1.79-fold and 1.69-fold increases in olive tail moment in H9 and BG01 cells, respectively (Figure 5A). Similar results as after irradiation were obtained upon treatment of hESCs, MCF7 and U2OS cells with the topoisomerase II poison etoposide (Figure 5B), which induces only DSBs. Thus, even under conditions that can promote G2 arrest, a sizable fraction of clastogen-induced DSB repair in hESCs appears to involve DNA-PKcs.

### Single DSB repair in terminally differentiated astrocytes

**Figure 6:**
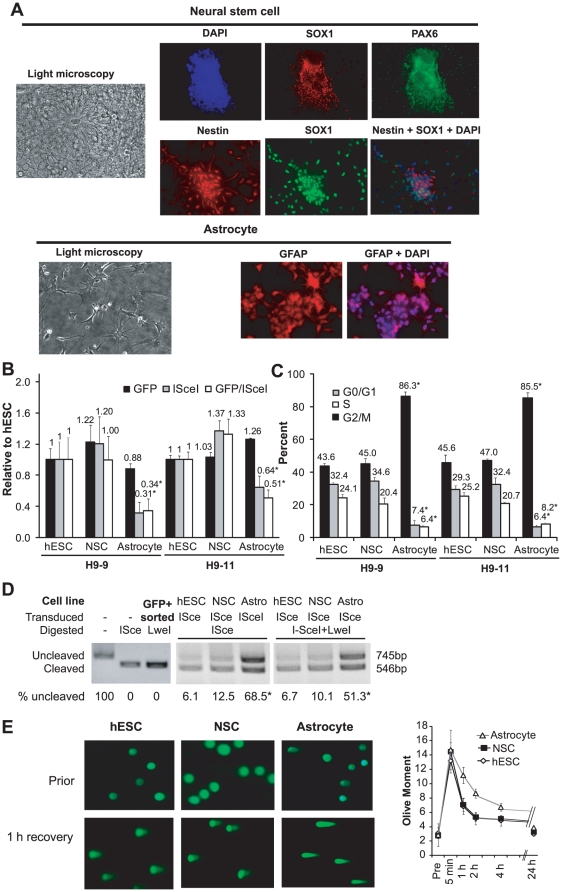
Repair at an I-SceI DSB in neural lineage cells. **A**. Light and immunofluorescence microscopy after differentiation of hESCs. DAPI indicates cellular nuclei. Neural stem cells are positive for SOX1, PAX6 and Nestin, while astrocytes are positive for GFAP. **B**. Percent GFP+ cells after GFP or I-SceI transduction relative to hESCs in two clones differentiated into NSCs and astrocytes. "GFP/ISceI" indicates the ratio of relative values. * indicates p<0.01 compared to hESCs from the same clone. Error bars indicated one standard deviation. **C**. Cell cycle distribution in two hESC clones and their differentiated NSCs and astrocytes. **D**. PCR amplification of the sequence flanking the I-SceI site from cells 96 hours after I-SceI transduction. PCR product was digested with I-SceI, LweI or both. "GFP+ sorted" cells were utilized as a control to demonstrate complete LweI cleavage. **E**. Comet assay at select time points after irradiation with 2 Gy. Examples are shown of cells quantified by comet assay either prior to or one hour after 2 Gy.

hESCs can be differentiated along committed lineages into either multipotent progenitors or terminally differentiated progeny. To determine the effects of neural differentiation on DSB repair, we differentiated H9 hESC clones harboring DR-GFP into neural stem cells (NSCs) and then into astrocytes (Figure 6A). Transduction efficiency in hESCs, NSCs and astrocytes was similar, based on the fraction of GFP-positive cells after infection with a GFP-expressing lentivirus (Figure 6B). After transduction of I-SceI, the percents of GFP-positive cells were similar in hESCs and NSCs (Figure 6B). However, the percent of GFP-positive cells after I-SceI transduction was reduced 36–69% in astrocytes compared to hESCs (Figure 6B; p<0.01).

Cell cycle arrest in G0/G1 could explain the low frequency of GFP-positive cells after I-SceI transduction. Cell cycle analysis demonstrated that approximately 86% of astrocytes are in G0/G1, compared with 43–47% of hESCs and NSCs (Figure 6C). As a result, the reduced rate of GFP positivity is likely to reflect the known dependence on NHEJ to repair DSBs in G0/G1. To determine whether this results in a greater frequency of imprecise repair, we PCR amplified the sequence flanking the I-SceI site, as above. While the percent of product resistant to I-SceI cleavage was similar in hESCs (6.1%) and NSCs (12.5%), 68.5% of product from astrocytes was resistant to I-SceI cleavage (p<0.01 compared with hESC or NSC) (Figure 6D). After double digestion with I-SceI and LweI (which cleaves all products except imprecise NHEJ), more than half of the product from astrocytes was resistant to cleavage, compared with 6.7% and 10.1% of product from hESCs and NSCs, respectively (Figure 6D).

To determine whether the preference for imprecise NHEJ among astrocytes is associated with either an increase or a decrease in the overall efficiency of repair, we performed the comet assay after IR, as above. Astrocytes retained more damage 1, 2 and 4 hours after irradiation (Figure 6E). Thus, terminal differentiation to astrocytes resulted in reduced overall repair capacity, with a preference for NHEJ.

## Discussion

We established a targeted reporter system for analyzing the effects of exogenous manipulations, *in vitro* differentiation, or genetic background on the repair of a single DSB in hESCs. Although other strategies have been used to target specific sites in pluripotent cells [19], [29], [35], *p84* ZFNs offer the desirable combination of very high targeting efficiency with a large (approximately 10 kb) construct and integration at a “safe” harbor locus [27]. Targeting DR-GFP to the same locus across multiple cell lines avoids potentially confounding locus-specific differences in repair. In fact, we observed a 20-fold difference in the frequency of HR based on site of integration. Considering these findings, targeting of DNA damage to the same site should be considered the “standard” for future studies that analyze repair across different genetic backgrounds and cell types.

Under the conditions we utilized, both hESCs and NSCs repaired a single DSB more precisely than either somatic cell lines or differentiated astrocytes. Of note, the 10.99% frequency of imprecise repair at an I-SceI DSB in hESCs is almost certainly an overestimation of the true frequency after a single break. Precise repair of an endonuclease-induced DSB, either by NHEJ or through HR using *SceGFP* on the sister chromatid, reestablishes the target site for the endonuclease. A reestablished site is a substrate for further iterations of cleavage and repair, so the frequencies of imprecise repair observed in studies like ours may capture multiple cleavage events within each transduced cell.

Differentiation of hESCs into astrocytes resulted in reductions in both the efficiency of IR-induced DSB repair and the precision of I-SceI-induced DSB repair. Adams et al. [36] analyzed the resolution of IR-induced γH2AX and RAD51 foci in H9 and BG01V hESCs, as well as differentiated NSCs and astrocytes. In contrast with our results, they reported a small but statistically significant increase in the number of γH2AX foci in hESCs, compared with astrocytes at various time points over the 20 hours after irradiation [36]. One possible explanation for the discrepancy is that focus resolution is an indirect marker of the kinetics of DSB repair. We utilized the comet assay to measure DNA repair, as assessment of the comet tail is a direct measure of DNA fragmentation. Consistent with our findings that HR is downregulated in astrocytes, Adams et al. reported that astrocytes have markedly lower levels of RAD51 protein and do not form RAD51 foci in response to IR [36].

The same group recently utilized a randomly integrated reporter to quantify NHEJ between two closely-spaced I-SceI DSBs in a single BG01V cell line [37]. Similar to our findings, the precision of NHEJ was reduced by differentiation of hESCs into astrocytes. In the BG01V cells cleaved by I-SceI, NHEJ was dependent on the Ligase IV co-factor XRCC4 but, in contrast with our results using IR or etoposide, only slightly affected by chemical inhibition or RNAi knockdown of DNA-PKcs [37]. One possible explanation is that DNA-PKcs plays a larger role in the repair of damage induced by IR or etoposide, compared with repair between I-SceI breaks. An alternative possibility is that inhibition of DNA-PKcs suppresses synapsis between DSB ends [38], [39]. Failure to synapse would allow DSB ends to repair with ends from other breaks, which is required in the two I-SceI break system utilized by Adams et al. [37].

Neural stem cells had similar repair efficiency and precision to hESCs propagated under similar conditions. Although both pluripotent and somatic stem cells proliferate when cultured *in vitro*, the latter are primarily quiescent *in vivo*. Milyavsky et al. recently reported that human hematopoietic stem cells readily undergo apoptosis when exposed to ionizing radiation, suggesting that these cells lack extensive capacity for DSB repair [15]. However, DSBs presumably form very infrequently in somatic stem cells *in vivo,* both because quiescent cells are not undergoing DNA replication and because of their relative metabolic inactivity. Thus, the ability of a somatic stem cell to orchestrate precise repair, rather than its efficiency at repairing multiple breaks, is likely to be the primary determinant of genomic integrity *in vivo*.

Within a multi-cellular organism, the necessity for faithful repair in any particular cell may differ across both cell lineage and stage of differentiation. A common hypothesis is that somatic stem cells must possess an extensive capacity for precise DNA repair [40], [41], [42], based on three arguments. First, somatic stem cells have long life-spans, during which they could accumulate potentially deleterious mutations. Second, many somatic stem cells undergo limited replenishment, suggesting that loss of stem cell function and/or viability would compromise organ function. Finally, somatic stem cells are the presumed cell-of-origin for many types of cancer, so the accumulation of mutations within these cells would be particularly dangerous to the entire organism. Similar logic would suggest that pluripotent stem cells, including hESCs, must also have highly efficient and precise mechanisms for repairing DNA damage. Mutations acquired within a pluripotent cell at the zygote or early blastocyst stage of development would necessarily be inherited within all or a fraction of the organism and potentially transmitted through the germline. Thus, faithful preservation of the genome in hESCs could be a highly evolved necessity.

The contrary argument is that the elimination of stem cells that incur DNA damage, rather than repair, offers a greater advantage. The hESC phenotype is presumably quite transient *in vivo* compared with overall lifespan, so evolutionary pressure for uniquely precise DNA repair within these cells seems unlikely. Instead, the high maternal “cost” of gestation and parturition could have selected for a low threshold for hESC elimination in the presence of DNA damage, especially highly mutagenic damage like DSBs.

In conclusion, *w*e have established a targeted reporter system for analyzing the effects of environmental, genetic and other manipulations on the repair of a single DSB in pluripotent and somatic stem cells, as well as their differentiated progeny. While hESCs cultured in the absence of feeder cells orchestrate more precise DSB repair than a panel of somatic cell lines, it will be important to determine whether changes in culture conditions can further improve the precision of repair in these and other pluripotent cells. Maximizing the precision of repair during *ex vivo* culture will reduce the likelihood that deleterious mutations, which could result in either diminished function or malignant transformation, develop within cellular therapy products. Conversely, strategies that depend on site-specific genomic editing could be enhanced by utilizing conditions that promote imprecise repair [43], [44].

## Materials and Methods

### hESC Culture and Neural Differentiation

The hESC lines H9 and BG01 were purchased from the National Stem Cell Bank (http://www.wicell.org) at passages 31 and 18, respectively. The cells were cultured on Matrigel (BD Bioscience) coated plates in mTeSR1 medium (Stem Cell Technology).

Neural differentiation was performed as described [45], [46]. Briefly, hESCs were treated with 1 mg/ml dispase (Stem Cell Technology) and split to poly-L-lysine/laminin (Sigma) coated dishes in N2B27 medium (1∶1 mix of D-MEM/F12 medium with 1x N2 supplement and Neurobasal medium with 1x B27 supplement (Invitrogen) supplemented with 100 ng/ml mouse recombinant Noggin (R&D Systems). The cells were split 1∶2 every 3–5 days. At approximately the 15^th^ day (passage 4) when cells appeared as small rosettes, a portion of the cells was collected and confirmed to express NSC markers. The remaining cells were continuously cultured in N2B27 medium with Noggin for 5 additional days and then split using TrypLE (Invitrogen) and changed to new N2B27 containing 20 ng/ml bFGF (Invitrogen) and 20 ng/ml EGF (R&D Systems). The cells were maintained in this condition for approximately 60 days (passage 14) with 1∶2 split every 5–7 days. The bFGF and EGF were withdrawn and the cells were cultured in N2B27 medium alone. After 7–10 days, cells were collected and assayed for astrocyte markers.

### Non-stem cell culture

293T, MCF7, U2OS, HCT116 and HeLa cells were obtained from ATCC. HEK293, MCF7 and U2OS cells containing untargeted DR-GFP and mESCs containing HPRT-targeted DR-GFP were provided by Maria Jasin (Sloan-Kettering Institute) and were previously described [7], [47], [48]. The human cell lines were cultured in DMEM supplemented with 10% fetal bovine serum, 0.1 mg/ml penicillin and 0.1 mg/ml streptomycin. The mESCs were cultured as previously described [7].

### Neutral comet assay

Double-strand DNA breaks were measured by neutral microgel electrophoresis using the CometAssay kit (Trevigen), following the manufacturer's instructions with some modifications. To prevent cleavage at heat- and/or alkali-labile related DNA strand breaks, all procedures were performed at pH 7.4 and ≤37°C. Briefly, exponentially growing cells were treated with IR, etoposide or mock and then incubated for various times at 37°C to allow for repair. After incubation, cells were cooled immediately to 4°C, and a 50 µL aliquot of cells (1×10^5^ cells/mL) was added to 500 µL of 0.5% low-melting agarose that had been boiled and then cooled at 37°C for 20 min. After mixing the sample, a 50-µL aliquot was pipetted onto an area of the CometSlide. The slide was incubated at 4°C for 10 min to accelerate gelling of the agarose disc, and then transferred to pre-chilled lysis solution for 30 min at 4°C. After lysis, the cells were first treated with ribonuclease A for 2 h, then with proteinase K for 48 h, both at 20°C. Following enzyme digestions, slides were subjected to electrophoresis under neutral conditions (100 mM Tris, 300 mM sodium acetate, pH adjusted to 8.5 with acetic acid) in a horizontal chamber at 1 V/cm) for 10 min at room temperature. The slide was fixed in ice-cold, 100% methanol for 5 min and then immersed in 100% ethanol at 20°C for 5 min and air dried. For observation, samples were stained with SYBR-Green (Molecular Probes) and diluted 1∶10,000 in 10 mM Tris-HCl, pH 7.5, 1 mM EDTA. Images of comets were visualized with a Zeiss Axio Observer.A1 after sample blinding. For DNA damage analysis, we used CometScore 1.5 software (TriTek Corporation) to compute the olive tail moment (OTM) for 50–100 cells from each sample. Each experiment (triplicate samples per group) was repeated at least three times.

### Cell cycle profile analysis

Cell cycle profiles were based on cell DNA content measured via flow cytometric analysis. Cells were rinsed with PBS three times, harvested after treatment with TrypLE (Invitrogen) and pelleted for 5 min at 1,250 rpm. The cells were resuspended in PBS, recentrifuged, fixed in 70% (vol/vol) ice-cold ethanol, and incubated on ice for at least 30 min. Immediately before analysis, the cells were pelleted by centrifugation for 5 min at 1,250 rpm and resuspended at 10^6^ cells/ml in PBS containing 50 µg/ml propidium iodide and 100 µg/ml RNase A (both from Sigma). The histograms of cell number versus DNA content were generated by flow cytometric analysis on a FACSCanto II (BD BioSciences). The data were analyzed using Modfit LT software (Verity Software House).

### Western blot analysis

Cells (10^6^–10^7^) were harvested and resuspended in 100 µl buffer I (10 mM Tris–HCl, pH 7.8, 200 mM KCl). The cell suspension was added to 100 µl of buffer II (10 mM Tris–HCl, pH 7.8, 600 mM KCl, 2 mM EDTA, 40% (v/v) glycerol, 0.2% (v/v) Nonidet P-40, 2 mM dithiotreitol, 0.5 mM phenylmethylsulphonyl fluoride and protease inhibitor cocktail (Sigma)). The mixture was shaken at 4°C for 40 min to promote cell lysis. The crude lysate was then centrifuged at 16,000×*g* for 10 min to remove cellular debris and DNA. Protein concentrations were determined using the BCA Protein Assay (Thermo Scientific). After the addition of 2-fold concentrated loading buffer, the samples (each with 30 µg total protein) were incubated at 95°C for 1 min and resolved by sodium dodecyl sulfate-polyacrylamide gel electrophoresis. Proteins were transferred onto nitrocellulose membranes (Schleicher & Schuell), incubated with blocking solution containing 3% powdered milk. Immunodetection of HA-tagged I-SceI was performed using anti-HA (MMS-101P Covance) mouse monoclonal IgG1 diluted 1∶200 in blocking solution, with an incubation at 4°C overnight. Anti-β-actin (AC-15, 1∶500; Sigma) was used as a loading control. Bands were detected with a fluorescent western detection (ECF) system (Amersham) and visualized by using the LAS 4000 Imaging Quantum Dots (Fujifilm).

### Plasmids and hESC viral infection

The reporter plasmid pHPRT-DRGFP and the I-SceI expression vector pCBASce were described previously [22]. The lentiviral backbone HFUW was obtained from the Trono laboratory. The I-SceI cDNA was cloned into the lentiviral backbone HFUW (courtesy of Eric Brown, University of Pennsylvania) using an EcoRI-EcoRI fragment to create the pHFUW-ISceI plasmid. The expression plasmid of zinc finger nucleases targeting AAVS1 and the AAVS1-GFP donor plasmid pGFP-AAVS1 were previously described [27]. The new donor plasmid p84-DRGFP was generated from pGFP-AAVS1 by isolating the 9.6 kb Sac1-Kpn1 fragment of pHPRT-DRGFP [7]. A linker containing an MluI site was inserted into pGFP-AAVS1 in place of the GFP gene and the SacI-Kpn1 DR-GFP fragment was cloned into the resulting plasmid after Mlu1/Kpn1 digestion.

The lentivirus were produced by transient transfection of 293T cells with pHFUW-ISceI and three package plasmids (pRSV-Rev, pRRE and pVSVG) using lipofecatmine 2000 (Invitrogen). After two days, supernatant was collected and used to transduce to 2×10^5^ cells in a single well of a 6-well plate along with 4 µg/ml Polybrene (Sigma-Aldrich) for 18 hours.

### DRGFP Reporter integration and AAVS1 gene targeting

For non-targeted integration, non-stem cell lines were transfected with circular DRGFP plasmid (4 µg) was transfected into 10^6^ non-stem cells by lipofectamine 2000 reagent (Invitrogen) in 1 well of a 6-well plate. After 24 hours, 1000 cells were split into a 10 cm dish with medium containing 1.5 µg/ml. For hESCs, the cells were treated with 10 µM rho kinase (ROCK) inhibitor (Calbiochem; Y-27632) 24 hours before the transfection. The transfection was conducted by Amaxa nucleofection (Nucleofector II, Lonza) using 2 µg of DRGFP plasmid and 2×10^6^ hESCs in 100 µl human stem cell nucleofector 2 solution with program A-023, according to the manufacturer's instructions. Cells were subsequently plated on Matrigel-coated plate in mTeSR1 medium supplemented with ROCK inhibitor for the first 24 h. Cells were selected in puromycin 0.5 µg/ml beginning 48 hours after nucleofection. Targeting was confirmed by PCR using the primers in Table 1.

For gene targeting to *p84* in non-stem cells, 6 µg of pZFN-AAVS1 and 20 µg of circular p84-DRGFP were co-transfected into 5×10^6^ non-stem cells by lipofectamine 2000 in a 10 cm dish. The following steps are the same as non-targeted cells. For hESCs, 1 µg of pZFN-AAVS1 and 5 µg of p84-pDRGFP were nucleofected into 2×10^6^ cells, as described above. To test the efficiency of gene targeting by NHEJ, pHPRT-DRGFP was first linearized by Sca1 and Kpn1 digestion. 5×10^6^ non-stem cells in a 10 cm dish were transfected with 6 µg pZFN-AAVS1 using Lipofectamine 2000. The next day, the cells were transfected with 20 µg of linearized DRGFP by electroporation (Gene Pulser Xcell System, Bio-Rad; 293 cell program, 0.4-cm cuvettes). Targeting was confirmed by PCR using the primers in Table 1.

### DNA repair and site-loss assay

Four days after I-SceI transduction, the percentage of GFP positive cells was quantified by flow cytometry on a FACSCanto II (BD BioSciences), as previously described [22]. Genomic DNA was extracted with the Puregene Core Kit A (Qiagen) and the site-loss assay and enzyme digestions were performed as previously described [22], [31]. Band intensities were quantified using ImageJ software (http://rsbweb.nih.gov/ij/). To amplify individual imprecise NHEJ events, the site-loss PCR product was digested with I-SceI and LweI and individual resistant clones were sequenced after TA cloning (Invitrogen). For each cell line, transductions, flow cytometry and site-loss were performed at least twice and averaged over three or more data points.

### Quantitative PCR for DRGFP copy number

Primer sequences are listed in Table 1. Quantitative PCR was performed using the CFX96 system (Bio-Rad). Samples (25 µl each) were prepared in triplicate in a 96-well reaction plate. Each reaction contained 20 ng genomic DNA, 200 nM of each primer, 12 µl water and 12.5 µl iQ SYBR Green Supermix (Bio-Rad) in a 2-step PCR: pre-heating at 95°C for 3 min and 39 cycles of amplification and quantification (10 s at 95°C and 30 s at 60°C). The reaction was monitored by melting temperature (10 s at 95°C and 5 s at 65°C) to ensure a single PCR product. For clones with random integration, primers amplifying the *APE1* gene on chr.14 (Table 1) were used as reference. For AAVS1-targeting, a sequence close to the integration site on Chr. 19 was used as reference. All primer pairs were confirmed to have linear amplification in the test range. The cycle threshold (Ct) of sample was normalized with the reference gene Ct. The DRGFP insert copy number was calculated according to the 2^ΔΔ Ct^ method [49], using control cell lines known to have a single integrated copy as reference [7], [11], [32], [47].

The sequence surrounding the I-SceI site is present in both the *SceGFP* and *iGFP* fragments of DR-GFP. Thus, we used a nested PCR strategy to first amplify only the intact *SceGFP* fragments. DNA was isolated 24 hours after I-SceI transduction and subjected to conventional site-loss PCR for 18 cycles. 1 µl of the product was utilized for qPCR, as described above. A reference PCR amplified a similar length sequence downstream of *iGFP*.

### Immunofluorescence imaging

Cells were fixed at room temperature with 4% paraformaldehyde for 30 minutes, washed with PBS, and then permeabilized with 0.2% Triton X-100 for 30 min. The cells were treated with blocking buffer (3% BSA + 5% FBS in PBS) for 2 h at room temperature and then incubated with primary antibodies and blocking buffer at 4°C overnight. Secondary antibody (1∶1000) was applied for 60 minutes after washing with PBS. The cell nuclei were then counterstained with DAPI (1 µg/ml). The cells were mounted with ProLong Gold antifade reagent (Invitrogen) and then visualized and captured using a Zeiss Axio Observer.A1 fluorescence microscope. Foci were quantified by manual counting after sample blinding.

The primary antibodies included mouse monoclonal antibodies against human nestin and rabbit polyclonal antibodies against GFAP (1∶200 and 1∶1000 both from Millipore MAB5326, AB804), rabbit polyclonal antibodies against human Nanog and rabbit polyclonal antibodies against human Oct3/4, rabbit polyclonal antibodies against human PAX6 (1∶100 from The Developmental Studies Hybridoma Bank, University of Iowa), goat polyclonal antibodies against human Sox1 (1∶100 from R&D System). For IR-induced damage foci, antibodies included mouse monoclonal antibodies against human phospho-histone H2A.X (Ser139) (1∶250 Millipore 05-636), rabbit polyclonal antibodies against human Rad51(1∶50 Calbiochem; PC130). Secondary antibodies used were donkey anti-rabbit IgG andgoat anti-mouse or anti-rabbit IgG with Alexa 488 or 555 (all from Invitrogen).

## Supporting Information

Table S1 **A comparison of imprecise NHEJ events from H9 hESCs and U2OS cells harboring DR-GFP.** Individual products were isolated from cells after transduction with I-SceI. Inserted nucleotides are in blue. Microhomology is underlined. Some sequences were obtained more than once (see annotation at the right of each row). I-SceI cleavage results in a 4 bp 3′ overhang.
(DOC)
