# Supplementary material for: Repair at Single Targeted DNA Double-Strand Breaks in Pluripotent and Differentiated Human Cells
Source: PLoS One. 2011 May 25;6(5):e20514. doi: 10.1371/journal.pone.0020514 (PMC3102116; doi:10.1371/journal.pone.0020514)
Supplement: Table S1 — A comparison of imprecise NHEJ events from H9 hESCs and U2OS cells harboring DR-GFP. Individual products were isolated from cells after transduction with I-SceI. Inserted nucleotides are in blue. Microhomology is underlined. Some sequences were obtained more than once (see annotation at the right of each row). I-SceI cleavage results in a 4 bp 3′ overhang. (DOC) [file pone.0020514.s001.doc]

Uncleaved sequence

| CAAGTTCAGCGTGTCCGGCTAGGGATAA CAGGGTAATACCTACGGCAAGCTGACCCT |
| --- |

hESC sequences (n=36)

#

| CAAGTTCAGCGTGTCCGGCTAGGGATAA TAA CAGGGTAATACCTACGGCAAGCTGACCCT – 1 |
| --- |
| CAAGTTCAGCGTGTCCGGCTAGGGATAA CA CAGGGTAATACCTACGGCAAGCTGACCCT – 1 |
| CAAGTTCAGCGTGTCCGGCTAGGGATA CAGGGTAATACCTACGGCAAGCTGACCCT – 2 |
| CAAGTTCAGCGTGTCCGGCTAGGGATAA T AGGGTAATACCTACGGCAAGCTGACCCT – 1 |
| CAAGTTCAGCGTGTCCGGCTAGGGATAA AGGGTAATACCTACGGCAAGCTGACCCT – 3 |
| CAAGTTCAGCGTGTCCGGCTAGGGATAA GGGTAATACCTACGGCAAGCTGACCCT – 2 |
| CAAGTTCGGCGTGTCCGGCTAGGGATAA CG GGGTAATACCTACGGCAAGCTGACCCT – 2 |
| CAAGTTCAGCGTGTCCGGCTAGGGATA GGGTAATACCTACGGCAAGCTGACCCT – 5 |
| CAAGTTCAGCGTGTCCGGCTAGGGA CAGGGTAATACCTACGGCAAGCTGACCCT – 1 |
| CAAGTTCAGCGTGTCCGGCTAGGGAT GTAATACCTACGGCAAGCTGACCCT – 3 |
| CAAGTTCAGCGTGTCCGGCTAGGGAT TAATACCTACGGCAAGCTGACCCT – 1 |
| CAAGTTCAGCGTGTCCGGCTAGGGATAA TACCTACGGCAAGCTGACCCT – 3 |
| CAAGTTCAGCGTGTCCGGCTAGGG TAATACCTACGGCAAGCTGACCCT – 5 |
| CAAGTTCAGCGTGTCCGGCTAGGGATA TACCTACGGCAAGCTGACCCT – 3 |
| CAAGTTCAGCGTGTCCGGCTAGGGA CT ATACCTACGGCAAGCTGACCCT – 1 |
| CAAGTTCAGCGTGTCCGGCTAGGGATA CCTACGGCAAGCTGACCCT – 1 |
| CAAGTTCAGCGTGTCCGGCTAGGGA TACCTACGGCAAGCTGACCCT – 1 |

U2OS sequences (n=40)

#

| CAAGTTCAGCGTGTCCGGCTAGGGATAA TAACATTCAAT CAGGGTAATACCTACGGCAAGCTGACCCT – 1 |
| --- |
| CAAGTTCAGCGTGTCCGGCTAGGGATAA CTTATTA CAGGGTAATACCTACGGCAAGCTGACCCT – 1 |
| CAAGTTCAGCGTGTCCGGCTAGGGATAA TTTA CAGGGTAATACCTACGGCAAGCTGACCCT – 1 |
| CAAGTTCAGCGTGTCCGGCTAGGGATAA T CAGGGTAATACCTACGGCAAGCTGACCCT – 1 |
| CAAGTTCAGCGTGTCCGGCTAGGGATAA CT AGGGTAATACCTACGGCAAGCTGACCCT – 1 |
| CAAGTTCAGCGTGTCCGGCTAGGGATAA TA AGGGTAATACCTACGGCAAGCTGACCCT – 1 |
| CAAGTTCAGCGTGTCCGGCTAGGGATAA TT AGGGTAATACCTACGGCAAGCTGACCCT – 1 |
| CAAGTTCAGCGTGTCCGGCTAGGGATAA T AGGGTAATACCTACGGCAAGCTGACCCT – 1 |
| CAAGTTCAGCGTGTCCGGCTAGGGATA CAGGGTAATACCTACGGCAAGCTGACCCT – 2 |
| CAAGTTCAGCGTGTCCGGCTAGGGATAA AGGGTAATACCTACGGCAAGCTGACCCT – 1 |
| CAAGTTCGGCGTGTCCGGCTAGGGATAA TTCCG GGGTAATACCTACGGCAAGCTGACCCT – 1 |
| CAAGTTCGGCGTGTCCGGCTAGGGATAA ATACC GGGTAATACCTACGGCAAGCTGACCCT – 1 |
| CAAGTTCAGCGTGTCCGGCTAGGGATAA GGGTAATACCTACGGCAAGCTGACCCT – 2 |
| CAAGTTCAGCGTGTCCGGCTAGGGATA GGGTAATACCTACGGCAAGCTGACCCT – 4 |
| CAAGTTCAGCGTGTCCGGCTAGGGA CAGGGTAATACCTACGGCAAGCTGACCCT – 1 |
| CAAGTTCAGCGTGTCCGGCTAGGGA GGGTAATACCTACGGCAAGCTGACCCT – 1 |
| CAAGTTCAGCGTGTCCGGCTAGGGAT GTAATACCTACGGCAAGCTGACCCT – 3 |
| CAAGTTCAGCGTGTCCGGCTAGGGAT TAATACCTACGGCAAGCTGACCCT – 1 |
| CAAGTTCAGCGTGTCCGGCTAGGGATAA TACCTACGGCAAGCTGACCCT – 3 |
| CAAGTTCAGCGTGTCCGGCTAGGGATA TACCTACGGCAAGCTGACCCT – 3 |
| CAAGTTCAGCGTGTCCGGCTAGGG TAATACCTACGGCAAGCTGACCCT – 5 |
| CAAGTTCAGCGTGTCCGGCTAGGGA CT ATACCTACGGCAAGCTGACCCT – 1 |
| CAAGTTCAGCGTGTCCGGCTA CTACGGCAAGCTGACCCT – 1 |
| CAAGTTCAGCGTGTCCGGCTAGGG GCTGACCCT – 1 |
| CAAGTTCAGCGTGTCCGGC GG GCTGACCCT – 1 |
